# Infant care practices and parent uptake of safe sleep messages: a cross-sectional survey in Queensland, Australia

**DOI:** 10.1186/s12887-020-1917-5

**Published:** 2020-01-21

**Authors:** Roni Cole, Jeanine Young, Lauren Kearney, John M. D. Thompson

**Affiliations:** 1 School of Nursing, Midwifery and Paramedicine, University of the Sunshine Coast, Sippy Downs, Queensland Australia; 2 Women and Families Service Group, Sunshine Coast Hospital and Health Service, Birtinya, Queensland Australia; 3 Paediatrics: Child and Youth Health, University of Auckland, Auckland, New Zealand

**Keywords:** Sudden infant death, SUDI, SIDS, Risk factor, Sleep-related infant mortality, Infant care practices, Safe infant sleep, Public health recommendations

## Abstract

**Background:**

Globally, the incidence of sleep-related infant mortality declined dramatically following the first public health campaigns seen internationally in the 1990s to reduce the risks of sudden infant death. However, Australian Sudden Unexpected Death in Infancy (SUDI) rates have plateaued with little change in incidence since 2004 despite two further public health safe sleep campaigns. This study aims to describe contemporary infant care practices employed by families related to the current public health SUDI prevention program.

**Methods:**

A cross-sectional survey of 3341 Queensland primary caregivers with infants approximately 3-months of age was conducted using the Queensland Registry of Births, Deaths and Marriages as a sampling frame. Surveys were returned either via reply-paid mail or online. Questionnaires explored prevalence of infant care practices and awareness of safe sleep recommendations. Univariable analysis was used to generate descriptive statistics for key variables.

**Results:**

Overall, only 13% of families routinely practised all six ‘Safe Sleeping’ program messages. More than one third (1118, 34%) of infants had slept in a non-supine sleep position at some time. Potentially hazardous sleep environments were common, with 38% of infants sleeping with soft items or bulky bedding, or on soft surfaces. Nearly half, for either day- or night-time sleeps, were routinely placed in a sleep environment that was not designed or recommended for safe infant sleep (i.e. a bouncer, pram, beanbag). Most babies (84%) were reportedly smoke free before and after birth. Sleeping in the same room as their caregiver for night-time sleeps was usual practice for 75% of babies. Half (1600, 50%) of all babies shared a sleep surface in the last two-weeks. At 8-weeks, 17% of infants were no longer receiving any breastmilk.

**Conclusions:**

The prevalence rates of infant care practices among this Australian population demonstrate many families continue to employ suboptimal practices despite Australia’s current safe sleep campaign. Strategic approaches together with informed decisions about pertinent messages to feature within future public health campaigns and government policies are required so targeted support can be provided to families with young infants to aid the translation of safe sleep evidence into safe sleeping practices.

## Background

Modifiable infant care practices are recognised as the most important factors parents and health practitioners can influence in order to reduce the risk of sleep-related infant mortality [1–3]. Sudden unexpected death in infancy (SUDI) is a term used to describe and classify deaths of apparently well infants for whom the cause of death is not immediately obvious and who would be expected to thrive; it includes sleep-related infant deaths classified as Sudden Infant Death Syndrome (SIDS), asphyxia, undetermined or ill-defined [4–6]. Infant mortality rates are widely recognised as key population and child health indicators [7, 8]. SUDI continues to be a major public health concern and remains the leading category of post-neonatal infant death in Australia [5].

The relationship between sleep-related infant mortality and modifiable infant care practices is well established [9–12], with 90–95% of sudden infant deaths associated with one or more recognised risk factors [13–15]. An Australian SUDI case review found that most SUDI occurred in an unsafe sleeping environment [5, 16]. Given individual vulnerable babies at risk of SUDI cannot currently be identified [4, 17] risk factors amenable to change have been the target of public health campaigns.

Globally, sleep-related infant mortality has fallen dramatically since the first public health prevention campaigns in the 1990s that focused on safe infant sleep and care practices including parent advice to avoid the prone infant sleep position. This advice is recognised as the main contributor to reduced SUDI rates. In Australia, the initial decline of 85% coincided almost immediately with Australia’s first national Safe Sleeping program in 1991 and is argued to be the only plausible explanation for this reduction (1.87 per 1000 live births in 1990; 0.3 per 1000 live births in 2003) [11, 18]. However, during the last 15 years, the Australian rate reduction has slowed and plateaued (0.3 per 1000 live births in 2017) [18]. This has not been the case for all Western countries with some countries, such as New Zealand and the United Kingdom, achieving continued declines in SUDI rates [4].

The development of Australia’s SUDI prevention public health programs and dissemination of the evidence-based Safe Sleeping messages is primarily supported by Red Nose (formally SIDS and Kids), a nationally recognised non-governmental organisation. The key recommendations promulgated are based on the modifiable factors parents and health professionals can influence the most. The current public health Safe Sleeping program contains six key messages: 1) Sleep baby on back; 2) Keep head and face uncovered; 3) Keep baby smoke free before and after birth; 4) Safe sleeping environment night and day; 5) Sleep baby in safe cot in parents’ room; 6) Breastfeed baby [19, 20].

Despite Australia’s national evidence-based health promotion program, inconsistencies exist amid the many forms of advice families receive, with each state and territory’s Health Department developing their own slightly modified policies and guidelines [17, 21–23]. This is especially so for those messages which have an emerging or developing evidence-base, such as shared sleeping, infant wrapping, and dummy use. Differences in interpretation of this evidence and/or policy/advice have led to a lack of consensus among researchers and policy makers as to exactly what the message should be. For instance, sharing a sleep surface remains a controversial topic with conflicting views on the associated benefits and risks with debate continuing in literature as to whether messages should convey a risk elimination (never share a sleep surface) or risk minimisation (reduce risk in all environments a baby may sleep whether intentional or not) approach. Many discussions challenge whether sharing a sleep surface itself poses a risk or if the risk is associated with the circumstances in which shared sleeping occurs [2, 24–26]. Due to challenges such as this, inconsistencies in resources exist both nationally and internationally, creating mixed messages within the community leading to confusion and potentially undermining key national public health messages.

Few studies have been undertaken in Australia to understand infant care practices that families use when caring for babies in home environments. In 2002, Young and colleagues benchmarked Queensland infant care practices and found many families employing suboptimal practices [27, 28]. Since this study, however, there has been no further investigation of practices despite two further national public health risk reduction campaigns launched by SIDS and Kids in May 2002 and the current campaign launched in 2012; resources were updated in April 2016 to reflect the restructured organisation of SIDS and Kids to Red Nose: Saving Little Lives ‘Safe Sleeping’ program (campaign messages remained unchanged) [4].

This paper presents findings from the 2017 Infant Care Awareness and Routines Evaluation among Queenslanders (I-CARE Qld) Study. The specific objectives of this paper were to determine contemporary infant care practices employed by Queensland families and identify consistency of practice with recommendations which underpin the current Australian ‘Safe Sleeping’ public health program [19, 20].

## Methods

A cross-sectional survey was conducted to explore infant care practices and sleep behaviours employed by families with young infants. Data were collected via a self-report questionnaire. The target population was Queensland primary caregivers with an infant aged approximately 3-months who was born in Queensland during April–May 2017. The three-month target age was chosen as it coincides with the period when infants are most vulnerable to SUDI [5]. The state of Queensland, Australia, has a population of 4.9 million and approximately 61,000 births annually [29]. Aggregate data from the Queensland Perinatal Data Collection (PDC) provided sociodemographic data for the target population.

The Queensland Registry of Births, Deaths and Marriages (the Registry) facilitated the state-wide distribution of the survey to home addresses of eligible families (*n* = 10,200) by using the Birth Notification register which provides the most comprehensive representation of the Queensland birth population. The Registry crosschecked the Birth Notifications register with national death data to remove infants who had died between birth and the time of survey distribution, to mitigate the risk of contacting recently bereaved families.

Correspondence included an introductory letter from the Queensland Registrar inviting families to participate in the project. Participant information provided to each family outlined the study purpose which was to understand current infant care practices used by families. Questionnaires could be completed either electronically via a unique weblink or by paper via reply-paid postage. To increase survey response rates, on behalf of the researchers, the Registry sent a reminder letter to non-responders 6 weeks after the initial distribution mail-out.

The survey tool was modelled on the 2002 Queensland Infant Care Practice Study [27] with the addition of contemporary questions, following critical analysis of recent studies, and synthesis of similar tools previously used to measure prevalence of care practices among primary infant caregivers. The questionnaire was structured to: collect infant and maternal demographics; describe infant care practices and sleeping routines; and explore caregiver awareness of the current national Safe Sleeping program. The questionnaire was piloted by 30 mothers of varying education and literacy levels to ensure the utility of the questionnaire and that questions were well defined, clearly understood and presented in a consistent manner; minor revisions were made.

Ethical approval to conduct this research was granted by the University of the Sunshine Coast Human Research Ethics Committee (S/17/1032). Participants received written information about the study informing them participation was voluntary and that the return of a completed questionnaire, either by post or electronic transmission, implied consent.

Online data was collected via Opinio [30], with paper questionnaire responses entered manually into Opinio on return. Data were analysed using IBM SPSS Statistics, Version 24.0. Univariate analysis was used to generate descriptive tabulations for key variables. Comparison of demographic characteristics compared those who took part in the survey to the target population. Categorical variables were compared using chi-squared statistic and continuous variables were compared using a t-test.

## Results

### Response rate

Of the 10,200 questionnaires distributed, 302 (3%) were returned to sender (that is not received by the intended recipient), 411 (4%) actively declined participation and 3341 were completed by families (total response rate 33% of eligible births). More families completed the paper survey returning the questionnaire via reply-paid postage (2439, 73%) than via the electronic survey link (902, 27%). Caregiver demographics of, and infant care practices used by, online and postal responders were similar (*p* > 0.05) with the exception of smoking, where maternal smokers were more likely to respond using a paper questionnaire than electronic survey, when compared to non-smokers (6.3% vs 4.2%, *p* = 0.02). For 97% (3247) of respondents the infant’s mother completed the survey.

### Demographics

Table 1 compares sociodemographic characteristics for mothers and infants for whom questionnaires were completed and the target population. Sample families were more likely to be reporting infant care practices for a first-born infant, be in a partnered relationship and be born in Australia. Families were less likely to identify as Aboriginal and/or Torres Strait Islander or be a younger mother. The median age of babies for respondent participants was 3.7 months (Interquartile range [IQR] 2.8, 4.1).

**Table 1:** Socio-demographics of sample population and target population

	Survey Participants n (%)	Target Population n (%)	*P*-value*
Maternal Characteristics	*n* = 3341	*n* = 10,131	
Age, years	(missing = 103)		
< 20 years	27 (0.8)	354 (3.4)	< 0.0001
20–29 years	1065 (32.9)	4432 (43.1)	
30–39 years	1982 (61.2)	5066 (49.2)	
≥ 40 years	164 (5.1)	435 (4.2)	
Parity	(missing = 51)		
Primiparous	1496 (45.5)	3079 (30.4)	< 0.0001
Multiparous	1794 (55.5)	7052 (69.6)	
Marital status	(missing = 57)	(missing = 15)	
Partnered [married/de facto]	3125 (95.2)	8120 (80.3)	< 0.0001
Single [never married, separated/divorced, widowed, not stated]	159 (4.8)	2046 (19.7)	
Country of birth	(missing = 57)	(missing = 1)	
Australia	2554 (77.8)	7386 (72.9)	< 0.0001
Overseas	730 (22.2)	2744 (27.1)	
Indigenous status	(missing = 74)		
Neither Aboriginal nor Torres Strait Islander	3205 (98.1)	9411 (92.9)	< 0.0001
Aboriginal and/or Torres Strait Islander	62 (1.9)	720 (7.1)	
Smoking status	(missing = 59)	(missing = 21)	
Smoked during pregnancy	135 (4.1)	1216 (12.0)	< 0.0001
Smoke-free during pregnancy	3147 (95.9)	8894 (88.0)	
Infant Characteristics	*n* = 3341	*n* = 10,287	
Sex	(missing = 29)		
Female	1552 (53.1)	4881 (47.4)	0.41
Male	1760 (46.9)	5406 (52.6)	
Birth weight, g	(missing = 147)	(missing = 19)	
< 2500	169 (5.3)	700 (6.8)	< 0.0003
2500–3499	1632 (51.1)	5444 (52.9)	
3500–3999	1012 (31.7)	3078 (29.9)	
4000–4499	323 (10.1)	938 (9.1)	
≥ 4500	58 (1.8)	127 (1.2)	
Gestation	(missing = 55)		
Term ≥37 weeks	3074 (93.5)	9400 (91.4)	< 0.0001
Preterm < 37 weeks	212 (6.5)	887 (8.6)	
Plurality	(missing = 74)		
Singleton	3220 (98.6)	9976 (97.0)	< 0.0001
Multiple	47 (1.4)	311 (3.0)	
Indigenous status	(missing = 61)		
Neither Aboriginal nor Torres Strait Islander	3166 (96.5)	9382 (91.2)	< 0.0001
Aboriginal and/or Torres Strait Islander	114 (3.5)	905 (8.8)	

*p-value for difference between participants and target population

### Practices related to Australia’s current Red Nose: Safe Sleeping program

The six recommendations comprising the current Australian national ‘Safe Sleeping’ program as outlined in the Red Nose Safe Sleeping guidelines [19, 20, 31] is used as a framework to present results. Table 2 summarises some of these key infant sleep practices across three-time categories (ever used, usual practice last 2 weeks, last night).

**Table 2:** Frequencies of key practices related to current safe sleeping program

	Ever used^a^	Usual practice last 2 weeks		Last night’s sleep
Position infant placed to sleep	*n* = 3307 (%)	*n* = 3310 (%)		*n* = 3308 (%)
Lying supine	3218 (97.3)	2746 (83.0)		2776 (83.9)
Lying prone	839 (26.8)	291 (8.8)		276 (8.3)
Lying on side	1118 (33.8)	273 (8.3)		256 (7.7)
Bedding/items in sleep environment	*n* = 3292 (%)	*n* = 3292^a^ (%)		
Blanket	2534 (76.6)	2258 (68.4)		–
Dummy	1586 (47.9)	1336 (40.5)		–
Pillow	718^b^ (21.7)	338 (10.2)		–
Soft toy	565^b^ (17.1)	352 (10.7)		–
Rolled towel/blanket	406 (12.3)	147 (4.5)		–
Position device/wedge	365 (11.0)	217 (6.6)		–
Doona/duvet	305 (9.2)	224 (6.8)		–
Beanie/hat/hoodie	274 (8.3)	55 (1.7)		–
Sheepskin	208 (6.3)	134 (4.0)		–
Cot bumper	183 (5.5)	160 (4.9)		
Infant nest	134 (4.0)	79 (2.4)		
Bed situation infant placed to sleep	*n* = 3303 (%)	*n* = 3308^c^ (%)	*n* = 3305^d^ (%)	
Bassinet	2379 (72.0)	1329 (40.2)	774 (23.4)	–
Cot	2003 (60.6)	1276 (38.6)	997 (30.2)	–
Double/queen/king bed	1777 (53.8)	367 (11.1)	258 (7.8)	–
Rocker/swing/bouncer	1541 (46.7)	17 (0.5)	357 (10.8)	–
Pram or stroller	1393 (42.2)	10 (0.3)	201 (6.1)	–
Infant carrier/baby sling	1215 (36.8)	–	107 (3.2)	–
Baby capsule/car seat	1140 (34.5)	3 (0.1)	86 (2.6)	–
Rug/playmat	699 (21.2)	–	70 (2.1)	–
Couch/sofa/armchair	669 (20.3)	6 (0.1)	134 (4.0)	–
Portable/travel cot	493 (14.9)	79 (2.4)	79 (2.4)	–
Co-sleeper device/nest on adult bed	299 (9.1)	73 (2.2)	37 (1.2)	–
Other bed type or sleeping surface	264 (8.0)	44 (1.3)	106 (3.2)	–
On a person	181 (5.4)	7 (0.2)	66 (2.0)	–
Mattress on floor	197 (6.0)	19 (0.6)	36 (1.1)	–
Clip on co-sleeper cot/crib	97 (2.9)	63 (1.9)	25 (0.8)	–
Single bed	61 (1.9)	12 (0.4)	11 (0.3)	–
Beanbag	57 (1.7)	2 (0.1)	11 (0.3)	–
Infant hammock	50 (1.5)	8 (0.2)	16 (0.5)	–
Water bed	6 (0.2)	0 (0.0)	0 (0.0)	–
Pēpi-pod	3 (0.1)	0 (0.0)	0 (0.0)	
Night-time room sharing		*n* = 3306^a^ (%)		*n* = 3311^a^ (%)
Own room (slept alone)	–	842 (25.5)		877 (26.5)
Room with mother	–	2445 (74.0)		2384 (72.0)
Room with father/partner	–	1873 (56.7)		1721 (52.0)
Room with other adult	–	13 (0.4)		16 (0.5)
Room with other children	–	211 (6.4)		244 (7.4)
Room with pets	–	198 (6.0)		185 (5.6)
Shared night-time sleep surface	*n* = 3296 (%)	*n* = 1600^ae^ (%)		
Double/queen/king bed	2286 (69.4)	1433 (89.6)		–
Couch/sofa/armchair	783 (23.8)	348 (21.8)		–
Baby has never shared sleep surface	772 (23.4)	–		–
Infant bed on adult bed	164 (5.0)	53 (3.3)		–
Cot/bassinet	154 (4.7)	29 (1.8)		–
Mattress on floor	139 (4.2)	56 (3.5)		–
Other bed type or sleeping surface	68 (2.1)	22 (1.4)		–
Single bed	57 (1.7)	14 (0.9)		–
Portable/travel cot	30 (0.9)	6 (0.4)		–
Beanbag	5 (0.2)	2 (0.1)		–
Pēpi-pod	2 (0.1)	1 (0.1)		–
Waterbed	1 (0.1)	1 (0.1)		–

^a^multiple responses allowed; ^b^any reported use of item ever; ^c^night-time sleep; ^d^day-time sleep; ^e^of those infants who shared a sleep surface during the last 2 weeks

#### Sleep baby on back

For 17% of babies a non-supine sleep position was usual practice in the last 2 weeks prior to survey completion; with similar proportions of babies being placed prone (8.8%) or on their side (8.3%). One in four infants had been placed prone for sleep at some time (see Table 2).

#### Keep head and face uncovered

The location of where baby was positioned relative to the end of the cot (reflecting uptake of the ‘Feet to bottom of cot’ guideline; a component of the ‘Keep head and face uncovered’ message) was explored for times when baby was placed in a cot during the last 2 weeks (whether or not this was usual practice). There were 2381 (72.3%) caregivers who reported that when baby was placed in a cot for sleep, the usual placement was with baby’s feet to the foot of the cot; 116 (3.5%) babies were placed in the cot with baby’s head towards the top of the cot. There were 173 (5.3%) babies who did not sleep in a cot at any time during the last 2 weeks.

Most parents reported the use of a baby sleep bag or commercially designed sleep swaddle (2448, 75%). Of those who had ever used a sleep bag or commercially designed sleep swaddle, 323 (14%) did not have fitted neck and arm holes.

#### Safe sleeping environment night and day

Bulky bedding and soft surfaces or items present in the infant sleeping place, along with bed types or sleep surfaces infants are placed to sleep on, are illustrated in Table 2. Sleep environments that contain bulky bedding or soft items (e.g. pillows, cot bumpers, soft toys, positioning devices, doonas) that can potentially increase the risk of suffocation or strangulation was usual practice for 1240 (37.6%) infants. Potentially hazardous bed situations, that is a sleep surface or bed type not designed or recommended for safe infant sleep (e.g. sofas, beanbags, infant rockers, adult beds), was usual for 703 babies for night-time sleep increasing to nearly one in two (1534, 46.4%) for day-time sleep.

Pillow use was routine practice for 338 (10.2%) babies. One in five babies (718, 21.7%) were reported to have slept on, or had within their sleep environment, a pillow at some time since birth; with 459 (13.9%) infants being placed to sleep on or with a pillow in the last 2 weeks. During the last 2 weeks 480 (14.5%) babies slept with a soft toy; this was usual practice for 352 (10.7%) infants. Having a soft toy in the sleep place at some time since birth had occurred for 565 (17.1%) of babies.

#### Sleep baby in safe cot in parents’ room

Sharing the same room as an adult caregiver at night-time was reported as usual practice for 2475 (74.9%) babies. For day-time sleeps 1515 (46.2%) infants slept in a room alone always or most of the time. Of those babies who did not usually sleep in a room alone at night, 329 (13.5%) were reported to always sleep in a room alone during the day.

For 2520 (76.9%) infants they had shared a sleep surface with another person at some time. When caregivers were asked if it was usually planned to share the sleep surface with baby, of those who had shared a sleep surface, 1443 (57.3%) indicated that it was not usually planned. Table 3 illustrates the reported frequency and duration of shared sleep when it does occur. Of the total sample, nearly half (1600, 49.6%) of the infants shared a sleep surface at some time in the last 2 weeks. Sharing a sleep surface with a mother was most common with 1544 (46.2%) and 792 (23.7%) babies had shared with their father or mother’s partner.

**Table 3:** Frequency and duration of usual shared sleep practice

	*n* = 3286 (%)
Frequency of shared sleep (missing = 55)	
Every night/normal routine	487 (14.8)
Most nights (4–6/week)	237 (7.2)
Some nights (2–3/week)	240 (7.3)
Occasionally (about 1/week)	1081 (32.9)
Rarely (less than 1/week)	398 (12.1)
Other	51 (1.6)
Baby has never shared sleep surface	792 (24.1)
Average time of shared sleep (missing = 105)	
Less than 1 h	745 (23.0)
1–3 h	958 (29.6)
4–6 h	322 (10.0)
More than 6 h	415 (12.8)
Baby has never shared sleep surface	796 (24.6)

#### Keep baby smoke free before and after birth

Maternal smoking during pregnancy was reported by 135 (4.1%) families (see Table 4). Most babies (2800, 85.3%) were described as living in a smoke-free household with no household members smoking. Respondents self-reported that 191 (5.8%) mothers had smoked cigarettes (any number) since having baby and 408 (12.4%) fathers had smoked.

**Table 4:** Infant smoke exposure

	*n* = 3341 (%)
Maternal smoking prenatal (missing = 59)	135 (4.1)
Maternal smoking postpartum (missing = 49)	191 (5.8)
Household smoking exposure (missing = 59)	482 (14.7)
Father/partner	408 (12.4)
Other household member(s)	57 (1.7)

#### Breastfeed baby

**Fig. 1:**
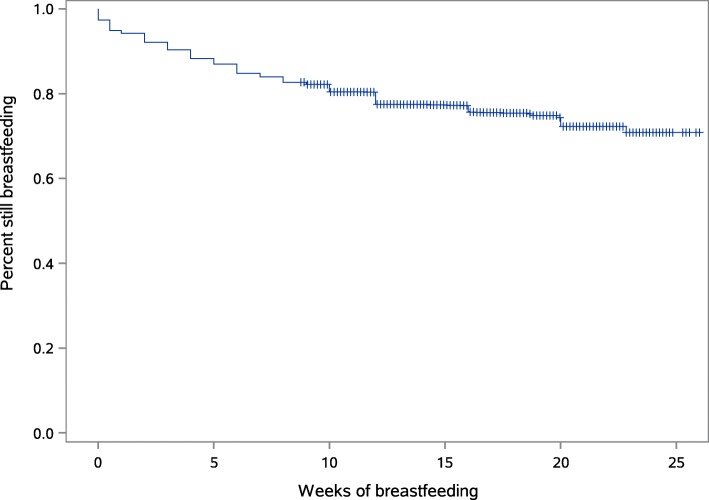
Kaplan-Meier survival curve of breastfeeding

Infant feeding practices are provided in Table 5. Of the 757 (22.8%) infants who did not receive any breastmilk over the 2 days prior to completing the questionnaire, 87 (11.5%) never received breastmilk after birth and a further 82 (10.8%) were less than 7 days old when they ceased receiving breastmilk. Figure 1 illustrates the age infants last received any breastmilk with 570 (17.2%) babies at 8 weeks of age no longer having any breastmilk; by 16 weeks of age a further 173 (6.9%) babies were no longer receiving breastmilk.

**Table 5:** Infant feeding practices

Feeding method	Feeding method when arrived home (or day 3 after home-birth)	Feeding method over last 2 days
	*n* = 3327 (%)	*n* = 3324 (%)
Breastmilk only	2567 (77.2)	2046 (61.6)
Infant formula only	154 (4.6)	757 (22.8)
Mixed (both breastmilk and formula)	606 (18.2)	521 (15.7)

### Family report of safe sleeping program implementation

Of the 3341 families who participated, only 426 (12.8%) reported sleeping routines and practices that were consistent with all six Red Nose ‘Safe Sleeping’ program messages; current at the time of the survey in 2017 [19, 20, 32]. Table 6 illustrates family reported uptake of the current ‘Safe Sleeping’ messages and supporting guidelines and advice outlined in the Red Nose mobile app and Safe Sleeping brochure [19, 20, 32].

**Table 6:** Frequency of Safe Sleeping program advice implemented in home environment practice

Red Nose: Safe sleeping messages and advice	*n* = 3341 (%)	Cumulative % following key messages
Safe Sleeping program: six ways to sleep baby safely		
Keep baby smoke free before and after birth (missing = 70)	2756 (83.7)	83.7
Sleep baby on back (missing = 31)	2746 (83.0)	70.7
Breastfeed baby (missing = 17)	2567 (77.2)	56.0
Keep head and face uncovered (missing = 162)	2148 (67.6)	43.0
Safe sleeping environment night and day (missing = 46)	1106 (33.6)	18.2
Sleep baby in safe cot in parents’ room (missing = 44)	1071 (32.5)	12.9
Key guidelines/advice provided within the six key messages		
Sleep next to parents’ bed [i.e. in same room as parents] (missing = 35)	2475 (74.9)	
Feet to bottom of cot (missing = 222)	2381 (76.3)	
No soft surfaces or bulky bedding (missing = 40)	2061 (62.4)	
Safest place for baby to sleep is in a safe cot* [i.e. avoid unsafe sleeping places/cot should meet current standard] (missing = 36)	1644 (49.7)	

*when sleep place reported as cot, it is assumed it is a safe cot meeting Australian Standard AS2172

## Discussion

Evidence-based risk reduction strategies known to reduce infant mortality underpin ‘Safe Sleeping’ recommendations, with key messages targeting modifiable factors which families can influence the most [23]. This study is the first investigation of infant care practices and the uptake of public health ‘Safe Sleeping’ messages in Australia for 15 years; a period which has seen two national public health campaigns launched. Findings demonstrate inconsistencies between current recommendations and the infant sleeping practices many Queensland families employ when caring for their young infant. In this study, only 13% of families reported they routinely practised all six of the current Safe Sleep recommendations when caring for their baby.

More first-time parents responded to this survey. Decisions families make in caring for their first child will often provide the foundations for future infant care practices and sleeping behaviours employed with subsequent children [33–35]. Therefore, where practices differ from current guidelines, this may indicate an underestimate of practices utilised by families in the broader population.

A significantly increased risk of sudden infant death is reported for babies who are placed prone for sleep (OR: 2.3–13.1) and for babies who are placed on their side and found prone (OR: 8.7) [36]. Despite over two decades of ‘back to sleep’ advice both nationally and internationally [12, 37, 38], non-supine sleep positioning persists with 17% of caregivers placing their infant to sleep in a prone or side sleeping position as their usual practice. However, this is less than that reported in other international studies where 26–33% reported non-supine positioning as usual infant sleep position choice [39, 40]. The dramatic reduction in SUDI incidence in the early 1990s has been directly associated with the widespread ‘Back to Sleep’ campaigns adopted at this time by many countries [2, 41, 42]. More than one-third of infants had been placed in a non-supine sleep position at some time since birth. These findings are similar to reports from other international studies (range 32–35%) [43–45].

Nearly 15% of families reported their infant lived in a household where at least one member of the household smoked. Maternal smoking during pregnancy was reported as 4%; this is double the rate (2%) reported in a 2013 New Zealand study [39]. Given the self-report nature of these studies it is likely that smoke exposure is indeed higher; given the rate of maternal smoking in pregnancy in the PDC data for the target population is 12%. Under-reporting of behaviours associated with stigma is common [3, 40, 46].

Smoking has recently been described as the most important current modifiable risk factor in reducing the risk of SUDI, with a population attributable risk estimated as high as 62% [2]. Since inception, Australia’s national public health campaigns to reduce sudden infant deaths have advised to keep baby in a smoke free environment [19, 20]. This same advice is supported globally in other international SUDI risk reduction campaigns [12, 37, 38]. It is estimated that if in-utero smoke exposure was eliminated, a third of sudden infant deaths could be prevented [47, 48]. Moreover, infants are more likely to be born prematurely and of low birth weight, further increasing infant vulnerability, when exposed to smoke during pregnancy [2].

Sleeping with head coverings, such as bonnets, hats, beanies and/or hooded clothing was reported as usual practice for 2% of infants, while 8% of babies had slept with head covering at some time since birth. Use of clothing and/or bedding in the sleep environment that may cover the infant’s face and/or head increases risk of airway obstruction and overheating [49, 50]. The results of two meta-analyses that examined the association between head covering and risk of SUDI suggest that over a quarter of these deaths may be prevented if the possibility of head covering was avoided [49, 50].

The advice infants be placed ‘feet-to-foot’; that is, positioning the infant with their feet to the bottom (foot-end) of the cot rather than with their head at the top or middle of the cot where they may be able to slip down under bedding contributes to recommendations in several countries [19, 20, 37]. We found 72% of Queensland families usually employ ‘feet to foot’ advice when babies are placed in a cot to sleep; slightly higher than a British population where 65% of infants were positioned ‘feet-to-foot’ [51].

The use of infant sleeping bags and commercially designed sleep swaddles have become increasingly popular, with 75% of families using a sleep bag or swaddle at some time. Current recommendations suggest the use of a safe infant sleeping bag removes the need for extra bedding in baby’s sleeping environment [19, 20]; loose bedding may pose a strangulation risk or cover baby’s face/head. However, care must be taken by the caregiver to ensure the sleeping bag is the correct size for the infant with well fitted neck and armholes, or sleeves (that prevents the infant slipping inside the bag), and does not have a hood [48]. Of those families who used a sleeping bag or commercially designed sleep swaddle, 14% did not use an infant sleep bag with fitted neck and arm holes.

More than one in three families usually placed their baby to sleep in a potentially hazardous sleep environment with infants reported to have soft or bulky bedding or objects, such as pillows, doonas, quilts, sheepskins, cot bumpers, positioning devices, rolled towels/blankets or soft toys, in their sleep environment. Similar findings were reported in a New Zealand study with homemade positioning systems reported to be increasing in prevalence [39]. Soft or bulky bedding or objects should never be placed under an infant to sleep, nor left loose in the infant’s sleeping space as they can increase the potential of suffocation, strangulation, rebreathing and entrapment [10, 36, 48]. Infants who sleep with soft bedding are reported to be at a five-fold increased risk of sudden infant death regardless of their sleep position and more than 20-fold if slept prone [48, 52].

One in four infants usually slept in a room alone for night-time sleep with nearly one in two sleeping in a room alone during the day. Studies suggest babies who sleep in a separate room to their caregiver, for both day-time and night sleeps, are at a greater risk of sudden infant death [36, 53, 54]. Sharing the same room as a caregiver permits close monitoring of the sleeping infant and allows for exchange of caregiver-infant sensory signals and cues providing protective and heightened infant arousal [55]. SUDI occurs more frequently in unobserved sleep periods where babies are more likely to be found with bedding covering their head or found prone when they were placed on their side to sleep, compared to babies who did not die and who slept in the same room as their caregiver [53, 54]. Room sharing with an adult caregiver is reported to reduce SUDI risk by up to 50% [36].

One in two infants were reported to routinely sleep on sleeping surfaces for night or day sleeps that are not recommended for safe infant sleep. While cots and bassinets were the most commonly reported bed type, adult beds were the second most commonly reported sleep surface usually used for night-time sleeps, with a rocker, swing or bouncer the second most common sleep surface for day-time sleeps. Internationally, guidelines recommend infants be removed from sitting products or devices such as bouncers, car seats, prams and baby swings for sleep [36]. Such devices are not designed as safe sleeping environments for babies; they may increase risk of airway obstruction due to chin-to-chest positioning or possible strangulation from straps [36].

To provide consistent messages to families, definitions for common terms relating to safe infant sleep need to be consistent. Unfortunately, this has not been the case for the terms *bed-sharing* and *co-sleeping.* These terms are often used interchangeably and are easily misconstrued. However, these terms are not synonymous. As several authors have highlighted [12, 26, 56] studies which use different criteria to define the same term create a confusing array of information that cannot easily be compared. This leads to further confusion among healthcare professionals and parents when interpreting and understanding safe sleep recommendations and the supporting evidence of such guidelines for infant sleep location. For example, co-sleeping is a term that has many definitions. It may be used to mean a sleeping arrangement where an infant sleeps on the same surface as another sleeping person [36, 57, 58]; or it may mean the infant sleeps in the same room as another without sharing a sleep surface [36, 59]; or it may mean a combination of both, that is, where the infant sleeps in close proximity (whether on the same or different surface) [36, 55]. Further confusion is added when the term bed-sharing is examined where a diverse array of definitions can be found among literature examining infant sleep location with some referring to bed-sharing as a sleeping arrangement where a caregiver and infant are both sleeping while sharing a sleep surface together [13], where others define it as taking baby into an sleep surface for feeding or comfort where sleeping is not necessarily intended but may occur [57, 58, 60]. Moreover, some definitions use the term co-sleeping within its definition of bed-sharing [57, 58].

The diversity and complexity of infant sleep location is truly remarkable and if confusion is highlighted among experts in the field, attention must be given to how this may be impacting practice through interpretation by families or information sharing provided by health professionals. Adherence to safe sleep recommendations increases when caregivers receive consistent messages from multiple sources with advice more likely to be followed when they understand the reasons and evidence underpinning a particular guideline [34, 61]. This consistency or lack thereof may represent a modifiable factor in promoting infant health and safe sleeping.

Even when parents do not intend to share a sleep surface with their baby it is common for parents to do so, even for short periods, with more than half of all families sharing a sleep surface when it was usually unplanned. This study found that more than one in four babies spent two or more nights a week sharing a sleep surface, with 23% sharing four or more hours. The difference between ‘planned’ care and reality is important to understand when safe sleep guidelines are developed, as recommendations should prepare parents for not only what they plan to do, but the reality of caring for a newborn. Careful consideration of the wording of public health recommendations and government policies is required when advising about infant sleep location to ensure strategies to mitigate risk in all sleep environments can be employed. Study findings are supported by observations of McKenna and McDade [55] who suggested infants rarely sleep in only one sleep environment, and therefore safety information for all sleeping arrangements should be provided to formulate successful public health messages. This approach acknowledges that parents may use strategies to reduce risk in circumstances where parents share a sleep surface with baby due to parenting preferences, cultural beliefs or unavoidable living circumstances, including instances where a parent may unintentionally fall asleep with a baby.

Infant mortality, specifically sleep-related infant deaths associated with suboptimal infant care practices, remains a universal priority. In a recent project to prioritise international SUDI research, Australian representatives identified ‘developing and evaluating new ways to make safe sleep campaigns more effective’ as a top research priority [62]. Findings from the I-CARE Qld Study provide vital information for stakeholders to move forward with this goal, assisting in the translation of current guidelines into contemporary, high quality, publicly accountable services, programs and policies that meet the needs of families to continue reducing sleep-related infant mortality.

Evidence generated from this study is important and unique, as it provides infant care practice data relating to the six-key messages in Australia’s current national Safe Sleeping public health program [19, 20]. Without contextualising infant care practices within the population in which most infants develop and thrive, greater limitations are placed on our ability to develop effective public health guidelines and parent support strategies to target and assist families most vulnerable to SUDI.

### Strengths and limitations

Study response rates were improved by providing reminder letters to invited families; an additional 23% (*n* = 762) of the final sample were received. This is an important participant recruitment strategy when planning data collection via postal invitation, particularly when intended participants are new families who have many demands for their attention. In addition, caregivers who were maternal smokers were significantly more likely to respond using the postal survey option compared to the electronic survey weblink; this has implications for future research into effective survey recruitment strategies in target populations experiencing greater vulnerability to SUDI.

Questionnaires were sent to the most comprehensive sample frame available to provide as close to a representative sample of the Queensland population as possible. However, it is unknown how those who chose to participate differ in practices to those who declined or did not respond. The demographics of participants indicates a more socially advantaged population; and as such, are more likely to be cognisant of public health messages. Therefore, results reported in this paper are likely to over-estimate the proportion of caregivers who follow safe infant sleep guidelines. Moreover, the present study was limited to one state of Australia. It would be desirable to conduct a larger national population-based study to assess prevalence differences among population groups and identify areas for targeted support nationally; especially when infant mortality is known to be higher among lower socioeconomic status groups and when Indigenous child mortality is twice the rate of non-Indigenous children [5].

A further limitation of this study, is the cross-sectional survey design using a self-report questionnaire, which limits infant care practices to a point in time, and subjects’ data to social desirability bias, likely providing an underestimate of less socially desirable behaviours. However, a cross-sectional design also makes such a study with a large population feasible and is consistent with reported studies that have measured infant care practices and sleeping routines within home environments for the well infant population [39, 51]. These factors, coupled with the demographics of the sample who responded may indicate an underestimate of the suboptimal practices employed by the wider community at large.

Despite these limitations, there is value in this study’s findings providing benchmarking of current practices against national recommendations; evidence for priority areas to develop and improve strategies to increase consistency of safe infant care practice; and a rationale for expanding research on infant sleep environments to more comprehensively explore the diversity and variations in infant care practices and sleep environments. Further, assessment of associations that influence family practice and the difficulties faced when implementing Safe Sleeping guidelines, along with parental decision-making processes used when deciding how they will care for and settle their infant to sleep, is required. The maternal and infant characteristics associated with suboptimal sleep routines and care practices that some families routinely employ warrants further analyses.

## Conclusion

The prevalence rates of infant care practices among this Australian population demonstrate that many Queensland families continue to employ suboptimal practices despite Australia’s current national public health campaign focused on reducing the risk of sleep-related infant mortality, launched in 2012. The use of non-supine sleep positions, not sleeping in the same room as an adult caregiver, and the use of soft bedding or additional materials in the infant sleep space were commonly reported by families; these practices are implicated with increased SUDI risk and likely contribute to preventable mortality.

This study provides an important benchmark from which to compare uptake and priorities for the current Safe Sleep recommendations that need further revision to establish more effective strategies in translating safe infant sleep evidence into practice, particularly for high risk target groups. Shared sleeping was highlighted with further investigation needed of ways to keep babies close to parents while supporting a safe sleep environment.

Without careful monitoring informed statements on the progress of reducing infant mortality cannot be made. Priority areas for future public health programs, education and research can only be realised when we understand contemporary infant sleep behaviours and practices that families employ in caring for their babies. Informed decisions about pertinent messages to be contained within future public health campaigns and government policies, along with practical evidence-based interventions that encourage and support families to implement safe sleeping practices is essential if we want sleep-related infant mortality rates to continue to fall.

## Data Availability

The datasets generated and analysed during the current study are available from the corresponding author on reasonable request.

## Abbreviations

I-CARE Qld: Infant Care Awareness and Routines Evaluation among Queenslanders
SIDS: Sudden Infant Death Syndrome
SUDI: Sudden unexpected death in infancy
